# Bearing witness: A grounded theory of the experiences of staff at two United Kingdom Higher Education Institutions following a student death by suicide

**DOI:** 10.1371/journal.pone.0251369

**Published:** 2021-05-12

**Authors:** Hilary Causer, Eleanor Bradley, Kate Muse, Jo Smith

**Affiliations:** 1 School of Allied Health and Community, University of Worcester, Worcestershire, United Kingdom; 2 College of Life, Health and Environmental Sciences, University of Worcester, Worcestershire, United Kingdom; 3 School of Psychology, University of Worcester, Worcestershire, United Kingdom; University of Toronto, CANADA

## Abstract

Wider networks of people are affected by a suicide death than originally thought, including those whose job-role brings them into contact with a death by suicide of another person. The impact of student suicide within United Kingdom (UK) Higher Education Institutions (HEIs) is unexplored and the experiences of staff members remain unknown. It is not known whether staff members have specific postvention needs following a student death by suicide. Any postvention support currently offered to staff members within UK HEIs lacks a context-specific evidence base. This study asked ‘How is a student suicide experienced by staff members within a UK HEI and what are the features of that experience?’ Staff members from diverse job-roles in two UK HEIs responded to a qualitative survey (n = 19) and participated in semi-structured interviews (n = 10). Data were transcribed and subjected to a constructivist grounded theory analysis. Participants’ experiences informed the development of a core category: ’Bearing witness’, which encompassed six further categories: ’Responding to a student suicide’; ’Experiencing a student suicide’; ’Needs and fears’; ’Experiences of support’; ’Human stories’; and ’Cultural stories’. The resulting grounded theory demonstrates how participants’ perceptions of impact are informed by their experiences of undertaking tasks following a student suicide within the community of their HEI. Processes of constructing perceptions of closeness to the student who died are evident amongst participants who did not know the student prior to their death. Tailored postvention support is required to respond to the range and complexity of HEI staff needs following a student death by suicide.

## Introduction

There were an estimated 793 000 suicide deaths worldwide in 2016, indicating an annual global age-standardised suicide rate of 10.5 per 100 000 population [1]. The likelihood of experiencing death by suicide of another person, in any given year, is approximately one in twenty (4.31%); the likelihood during a lifetime is one in five (21.83%) [2]. Far wider networks of people are affected by a suicide death than originally thought, extending beyond family members and close friends [3,4]. Perceptions of psychological closeness to the person who died, rather than relationships to the deceased, appear to affect the level of impact [5]. Higher levels of impact have been observed amongst close family members, wider family, social and work networks, but also amongst those in contact with the decedent due to the nature of their death, for instance first responders [6]. Perceptions of greater closeness and impact are related to higher incidences of depression, anxiety, Post Traumatic Stress Disorder (PTSD), and prolonged grief [6]. It is, therefore, important to expand our understanding of how those in wider networks are impacted by suicide.

Amongst the broader network of individuals impacted by suicide are those who experience a suicide death connected to their place of work or job-role. In some professions the likelihood of this experience is high. In a survey of 120 GPs, 86% encountered at least one patient suicide in the previous ten years [7]; of 247 consultant psychiatrists, 68% experienced a patient death by suicide [8]; 55% of 531 psychiatric nurses encountered at least one patient suicide [9]; of 89 psychiatric trainees, 43% experienced one or more suicides [10]; for teachers, nearly 36% of a sample of 145 were exposed to at least one student suicide [11]; and, in a sample of 697 social workers, 33% experienced exposure to suicide in their job role [12]. In comparison to the general population [1], those working in health, social care, and educational environments experience an increased risk of exposure to death by suicide during their working lifetime.

The effects of such rates of exposure have been evidenced amongst health, social care, and education practitioners [13]. These have included feelings of professional doubt, fear of legal consequences [14], a sense of responsibility for the death [15], emotional turmoil and stress reactions [14], and severe distress [16]. For some mental health professionals, severe and persistent post-traumatic responses such as intrusion, avoidance, and hyper-arousal have been reported at levels within a clinical range [17]. Teachers reported that they felt impacted in their personal life (76% of 145 teachers) with experiences of low mood and poor sleep, and their professional life (86%) by heightened awareness of suicide risk, increased use of existing protocols, decreased self-confidence, and changes to practice when encountering potentially suicidal students [11]. Amongst health professionals, the severity of response to a patient death by suicide has been linked to an interplay between sense of closeness to the deceased; level of exposure to the suicide; and experiences of support and training [18]. Feelings of responsibility for the death and concerns for the bereaved family are also thought to influence adjustment and coping following a patient suicide [15].

### Impact of suicide on first responders and those first on the scene

First responders typically come into contact with a suicide death with no knowledge of the deceased. In a sample of 61 US firefighters, the average lifetime exposure to suicide was 13.1 [19]. Cumulative exposure to both attempts and death was positively correlated with suicidal behaviour amongst exposed firefighters [19]. Female firefighters exposed to suicide during their careers experience more severe psychiatric symptoms and increased suicide risk compared to counterparts with no exposure [20]. High levels of occupational exposure to suicide for law enforcement officers was significantly associated with PTSD and other mental health symptoms [21]. A systematic review of the impact of suicides and other critical incidents involving railway personnel found some workers experienced diagnosable traumatic reactions following an incident; whilst others, experienced ongoing stress and fatigue [22]. Contact with the corpse and perceptions of victim vulnerability were amongst factors that increased negative reactions [22]. The impact of responding to suicide deaths for ambulance staff includes ongoing salient memories of events they have witnessed; being haunted by events; interference with sleep; feelings of personal distress and vulnerability [23]. Ambulance staff are often the first at a scene and may become involved with tasks that are beyond their usual job-role, including negotiating with a person in crisis; informing family of the death of a loved one; preserving a potential crime scene; and exposure to intense reactions of bereaved individuals [23]. A focus group study of 35 first line responders revealed they experienced feelings of inadequacy when faced with emotional bereavement reactions; felt unable to offer solutions due to self-doubt and a lack of guidance; and used emotional shutdown as a means for preservation [24]. Despite these challenges, first responders play a critical role for those who are bereaved by suicide during the immediate crisis stage of the aftermath [25]. Their behaviour can reduce risk of contagion and promote healing for family and others who are present at a suicide death [25].

### Impact of suicide within Higher Education Institutions (HEIs)

At least 95 HEI students died by suicide in England and Wales in the year ending July 2017 [26]. The impact of student suicide on individuals within university or college communities remains under-explored. Whilst it is known that students do experience exposure to suicide deaths [27], there are no known studies that report on exposure to, or impact of, student suicide amongst student peers or staff members in HEI settings. The impact of a student death by suicide on HEI staff members is unknown.

### Postvention support in HEIs

Postvention is a term used to describe things that happen to support a person after a suicide attempt or a death by suicide [28]. Andriessen [29] (p. 43) describes postvention as ‘activities developed by, with, or for suicide survivors, in order to facilitate recovery after suicide and prevent adverse outcomes including suicidal behaviour’. There is an increased risk of suicide amongst those who are exposed to suicide [30,31] and postvention support may lessen that risk [32].

Currently, postvention provision typically comprises support groups and psychological support. It is delivered through a range of in-person and remote platforms by a diversity of providers, usually aimed at meeting the emotional needs of family members and those closest to the deceased [33]. There is currently little recognition that staff members in HEIs may require postvention support. Many staff are often providers of support to others, rather than acknowledged as potentially needing support themselves [34]. When support is provided, HEIs can refer to public health and specific HEI guidance to help inform postvention provision [34–38]. However, there is inconsistent evidence for the effectiveness of postvention support and positive indicators require further methodologically robust research [33,39,40]. Furthermore, as the impact of suicide within HEIs is under-researched, specific postvention needs remain unknown. As other groups of workers experience diverse and impactful affects when their job-role brings them into contact with a death by suicide [13–25], it is pertinent to understand what kinds of impact, if any, are experienced by HEI staff members as a group. This would inform current models of postvention provision within HEIs by providing a context-specific evidence base. This study therefore focuses specifically on the experiences of HEI staff after a student death by suicide to inform the need for, and development of, appropriate, targeted, postvention support for a group of workers who are currently unrecognised as needing support, whilst being tasked with providing postvention to others.

## Research question

The study aims to critically examine the experiences and perceptions of a cross section of staff working within UK HEIs where a student suicide has been a recent event by asking: How is a student suicide experienced by staff members within a UK HEI and what are the features of that experience?

## Methods

Data were collected by mixed methods, utilising an electronic survey and semi-structured interviews. Given that there is a death of literature addressing this topic, an inductive, exploratory approach was appropriate. As such, a qualitative approach was selected for this study in order to explore the experiences, perceptions and meaning-making processes of the participants. As such, open text e-survey data and interview data were subject to a constructivist grounded theory analysis [41]. Grounded theory was particularly suited to this previously un-researched population as it is a theory generating method that provides findings that translate helpfully into policy or practice guidance [41].

### Ethics

Ethical approval for this study was given by the lead author’s institutional Health and Science Research Ethics Committee (REC no: SH17180008-R). Given the topic sensitivity, attention was paid to ensure anonymity of participants, institutions, and students who had died by suicide. The consolidated criteria for reporting qualitative research (COREQ) provided a guiding framework in the reporting of this study [42].

### Participants

Five potential HEIs were invited to become a recruitment site, two of which consented. Inclusion criteria for HEIs was that a full-time student at the HEI had died by suicide no longer than two years and not more recently than 9 months prior to data collection. Participants were HEI staff members, across diverse job-roles, who perceived themselves to have been impacted by a student death by suicide at a UK HEI (Table 1). Participants were invited to respond to the survey. At the end of the survey, they were offered an opt-in to the interview phase of data collection. Nineteen staff participated in the survey, 10 of whom participated in the follow-up interview. Eleven of the participants were female, eight were male. Participants had been in their job roles for between one and a half and twenty-four years. They ranged in age from 30 to 70.

**Table 1:** Study participants by gender, job-role, and site of recruitment.

		Site 1		Site 2	
		Survey	Interview	Survey	Interview
**Gender of participants**	Male	6	2	2	1
	Female	6	5	5	2
	**Total**	**12**	**7**	**7**	**3**
**Job-role of participants**	Executive Staff	1	1		
	Student Facing Staff*	5	3	1	1
	Facilities Staff**	3	3	2	
	Academic Staff	3		4	2
	**Total**	**12**	**7**	**7**	**3**

* Student Facing Staff: Student support services, counselling and wellbeing; student union staff.

** Facilities Staff: Accommodation, maintenance, domestic, security staff.

### Consent

Consent was obtained from a senior executive staff member at each HEI site for participants to be recruited. Consent was also obtained from staff participants before they completed the survey. Those who volunteered to participate in the interview study were consented prior to interview.

### Data collection

Data was collected via open text e-survey questions and semi-structured interviews. The e-survey collected demographics (gender, age, and job-role) and open text data pertaining to HEI staff perceptions of impact, experiences, and needs following a student death by suicide. The survey was disseminated by a key contact at both HEI sites to staff who had been involved with a student who had died by suicide or who had responded to a student death by suicide. The interview topic guide (S1 Table) was designed to utilise open-ended, non-judgemental questions, followed by further exploratory prompts [41]. Topics included participants’ experiences and perceptions of events following the student death by suicide and roles or tasks undertaken following a student death by suicide. Two interviews were conducted by telephone, eight were conducted on the participant’s campus. Interviews lasted between 38 minutes and 1 hour 15 minutes. Interview data was digitally recorded onto a password protected memory card for later transcription by the researcher.

### Data transcription

Data were transcribed verbatim, removing identifying data including names, specific job-roles, location details, identifying HEI details, means used, and details of the student who died. Interviews were transcribed in batches; interviews 1–5 were transcribed and initial coding undertaken; interviews 5–10 were transcribed at second stage coding.

### Data saturation

Glaser and Strauss [43] define theoretical saturation as being the point at which *‘no additional data are being found whereby the sociologist can develop properties of the category’* (pp61). However, Low [44] acknowledged a researcher may never know if there would be further new information in subsequent interviews or data and may be unable to confidently claim that theoretical saturation was achieved. A more pragmatic definition of theoretical saturation was adopted for this study [44]. The concept of theoretical saturation is informed by the quality of the data collected, and depth and rigor of the analytic process. In this study, the analysis of data was considered complete when there was nothing further to add to the categories that had been developed.

### Data analysis

A number of analytic tools were used throughout the process of grounded theory analysis [41], including constant comparison [43], memo-writing [41], and clustering [41].

### Analytic process

The analytic process included two stages of coding, initial line-by-line coding, followed by focused coding prior to the construction of categories and a core category [41]. All coding was undertaken by hand on printed transcripts. An overview of the analytic process can be seen in Table 2.

**Table 2:** Stages of code and category construction in grounded theory analysis.

Stage of Analysis	Data source	Process	Outputs
Stage 1	Interviews 1–5	Line by line coding using gerunds. Collapsing codes into each other to develop focused codes.	569 Initial Codes 32 groups of Codes
Stage 2	Interviews 6–10	Coding using code group headings. New codes developed where data did not ‘fit’. Groups of codes from interviews 1–5 and new codes collapsed into each other to generate focused codes.	158 Codes that sit within 23 groups of codes
Stage 3	Interviews 1–10	Ongoing comparison of transcripts, codes and groups of codes. Memo-writing and clustering to develop focused codes and categories.	63 Focused Codes 11 Categories
Stage 4			
Stage 5	Open text data from survey incorporated	Data coded using focused codes. Data comparison to confirm categories. Memo-writing and clustering used to check and broaden categories.	11 Categories
Stage 6	All data	RE-naming of some categories. Development of a final set of categories and sub-categories and the construction of a core category from an existing category.	One core Category Six categories Four sub-categories
Stage 7		Construction of a grounded theory.	Diagrammatic representation of the theory

An iterative process of checking, re-visiting, comparing, and further memo-writing ensured that, as codes, categories, and theory were constructed, they remained close and true to the data. Participants’ stories embedded in the data were developed through the researcher’s processes of applying research questions, ideas, and perspectives to construct a theoretical explanation of their perceptions, experiences, and needs. The resulting grounded theory is presented below.

## Findings

**Fig 1:**
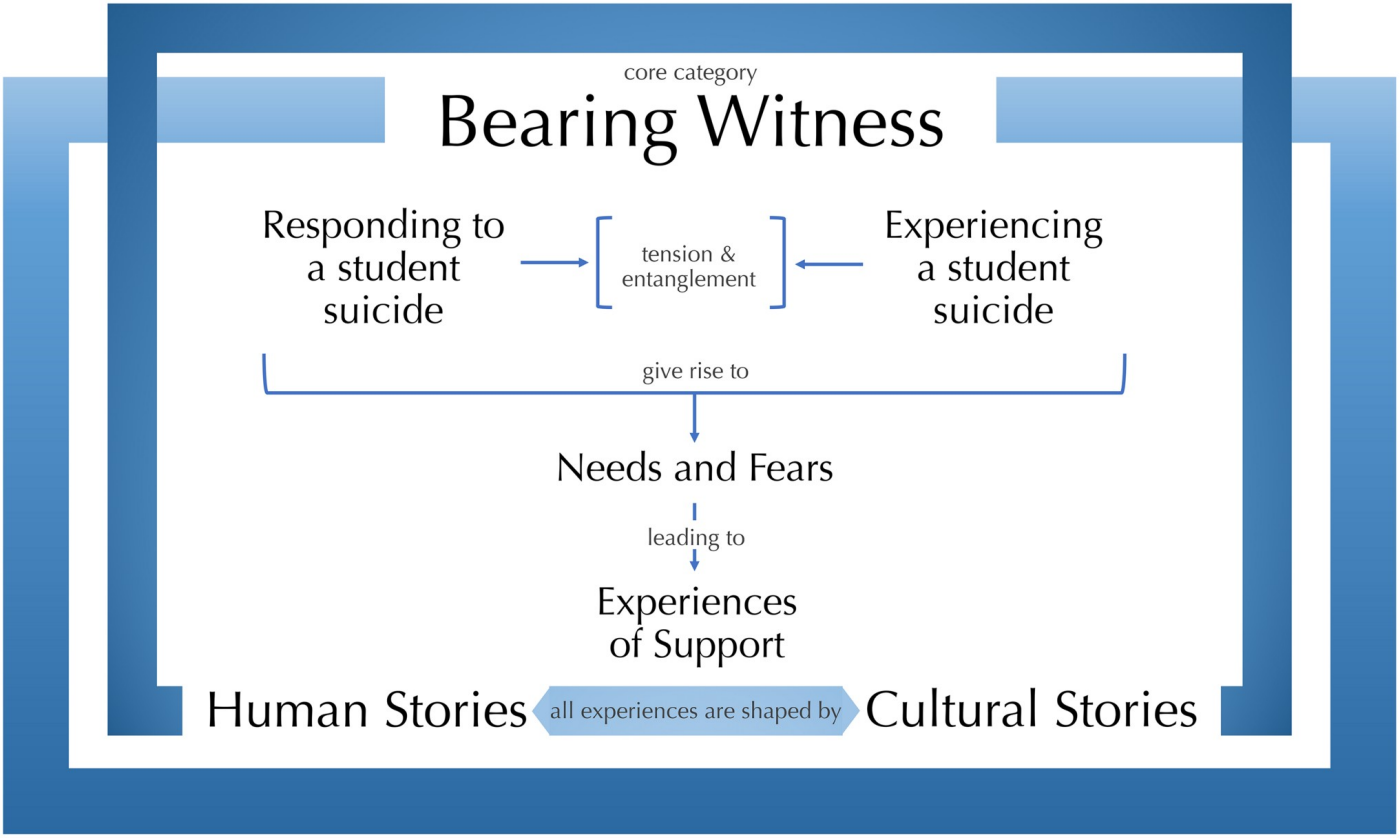
A grounded theory of the experiences of staff in UK HEIs who are exposed to a student death by suicide.

Analysis resulted in the construction of a core category ‘bearing witness’ that encompasses participants’ experiences in six categories and four sub-categories, which incorporate sixty-three focused codes. The relationship between the focused codes, sub-categories, categories and core category is illustrated in tabular format in S2 Table. A grounded theory was constructed (Fig 1) that describes processes and relationships experienced by staff members between and across categories.

### Core category: Bearing witness

It is in bearing witness to a student suicide that all subsequent experiences are shaped; and through the acts of responding and the processes of experiencing that staff members do bear witness. From the beginnings of the experience, the discovery of a body, or hearing news of a death, the staff member becomes witness to the death by suicide of a student. The term ‘bearing’ is fitting as the staff member experiences a sense of having to carry the weight of knowledge and realisation of the implications. There is a sense of being burdened by a sad and horrible reality:

‘*It’s terrible to think a young adult who I interacted with on a weekly*, *if not daily basis*, *was struggling to such an extent that they felt that suicide was their only option*.’

Academic staff.

There is no way for staff to walk away from this reality. It is their job to respond. The bearing is non-negotiable, it is unavoidable.

Being a responder and the ways in which staff members respond are made more awful, urgent, or challenging when a student has felt that their only choice was to end their own life and no one was present to stop them. There seems to be an amplification to the nature and purpose of the response that would not be present in other contexts. There is an urgent desperation present in staff accounts of how they responded that is not about the act of responding, but about responding within this context. There is a sense that, if we failed this student, then we absolutely must get this bit right:

‘*I mean I was actually in the end I was quite bossy*, *cos it was like*, *right this is what we need to do*’

Facilities staff.

As staff witness the body of the deceased student, the place of death, the student’s flat, or have contact with the student’s housemates, the student starts to become a person. Staff start to see, witness, or learn about the student. As this happens, the awfulness grows and amplifies the sense of needing to respond now, quickly, professionally, empathically, discreetly, and as fully as possible. A weight of responsibility is palpable throughout the accounts:

‘*you sit and reflect don’t you*, *because … you do really feel the level of responsibility*, *you know*, *students are*, *when their parents hand them over in September*, *they expect them to come home at Christmas* …’

Senior leader.

This was sometimes in the context of not knowing how to get it right, due to lack of previous experience together with multiple unknowns, including what parents would want us to do? It is a precarious tightrope for staff, who try to keep their balance by constantly checking, communicating, and meeting with each other, referring to guidelines, seeing what others are doing, and taking guidance from the behaviour and responses of others within and beyond the HEI.

In and through these activities and responses, staff bear witness not only to the death of a student, but to the impact of that death, the involvement of emergency services and other agencies, the presence of media who urgently wish to ‘bear witness’ on behalf of the public, the reactions of housemates, and the potential impact that this may have on student peers. Some staff bear witness to the responses and behaviours of parents and family members as the news is broken to them; witnessing their pain, horror and disbelief; or equally, witnessing their strength, containment, and ability to face the world. Wider communities throughout the University such as course groups or club mates will also learn the news. It is staff members who inform them of the suicide, witness their reactions, support and guide them through the process of learning that their peer, friend, or team mate has taken their own life:

‘*It was my responsibility to inform the student’s peers what had happened and manage their needs*. *In particular making sure they knew who to talk to and how to access counselling and bereavement services*… *It’s vaguely within my job description*, *though not explicit*. *I’m in charge of the programme so I had to perform certain duties as part of my duty of care towards the students*.’

Academic staff.

Bearing witness also happens in the being of people; processing of things; construction of ideas; perceptions of closeness; feeling and reflecting on personal responses to what has happened. It happens, for staff, in recalling what they had to do, how they did it, what they saw, what they said, what others did, saw and said. Having just experienced it all, the immediate fear of somehow being responsible, of having missed a sign, got something wrong, failed to do their job, to keep a student safe, to keep other students safe, starts to emerge:

‘*but there’s actually no guarantee that there’s not going to be three or four [more suicides] coming up and*, *and that would floor us I think*, *cos then you just feel*, *one the awfulness of it and then two you feel quite helpless*, *you know*, *that the stuff you’ve done [has made no difference]*’

Academic staff.

Needs emerge for staff, but they may feel selfish, self-indulgent, or superfluous. Needs may also generate a place to position blame or offload responsibility, ‘I wasn’t trained for this’; ‘the university isn’t looking after me’; just as ‘they’ let that student die, so ‘they’ are leaving me alone with the pain that has come from that death.

‘*Management in many ways treated the death of students as (a) crisis to be managed and forget that the staff closest to the student are not just their job-role but also people impacted by the event*. *I therefore felt unsupported by my employer in terms of giving me the space I needed to make sense of what had happened*. *I felt like I was a tool allowing more senior managers to demonstrate their management abilities to even more senior managers*. *This felt vulgar and upsetting given what had just happened*.’

Academic staff.

Experiences of support are influenced by re-visiting what has happened, re-living or re-feeling, also off-loading and feelings of relief and subsequent guilt about that sense of relief. For some staff who are witness to the aftermath of a student death by suicide, the bearing continues long after the death.

‘*This worry about other students continues to this day and impacts most on my life*. *There is always a concern that anything you do might affect a student*, *so it impacts on student interactions*, *teaching practices and generally feeling comfortable in the role*.’

Academic staff.

### Responding to a student death by suicide

‘Being the responder’ describes multiple ways staff respond by doing tasks immediately and following a death by suicide:

‘*[the student] was cold and obviously checking for a pulse and there was nothing there*, *yeah [pause] [nervous laugh] bit of a bad thing to find*’

Facilities staff.

Panic, nervousness, and fear might be present, actions appear driven by a need to respond quickly, professionally, and to manage the event:

‘*lots of students in that same block could see what was happening*, *maybe they didn’t know what had happened*, *so we had to say there’s been a death and we can’t tell you anything about it but we don’t want you*, *you*, *you don’t need to be concerned about yourself*, *you’re trying to reassure them*, *you can’t really tell them anything*, *you can’t tell them who it is even though they probably know who it is*’

Student facing staff.

Attention appears focused on tasks, the needs of others, the busy-ness and the necessity to respond efficiently, get it right, be thorough, to notice and meet needs. There are different kinds of tasks that staff may take on due to their job-role or because they are available at the time:

‘*everyone has a different role*, *so we don’t*, *it’s not like it’s just you having to deal with this terrible thing that has happened*, *so I think that is helpful*.’

Student facing staff.

‘Ways of responding’ describes ways staff deliver the response. It includes aspects such as adopting a mindset of ‘doing the job’; strategies for working with others; working with procedures; taking on roles and responsibilities:

‘*well I think for the first few days*, *it’s just er get on with the job*’

Student facing staff.

Some of these ‘ways’ are rooted in policy or experiences of practice, in hierarchy of job-role and responsibility within the HEI; others are rooted in external frameworks such as emergency services and coroners’ responses, procedures, and roles:

‘ *One of the things I reflected on later*, *cos it was fraught and it was challenging*, *one of the more challenging periods I’ve been through with work was that the amount of holding and containing and responsibility that a small number of people had … which is fine and it fitted with our roles and what our skills were*, *but it did feel from an institutional level that they would let us get on with it*’

Academic staff.

### Experiencing a student death by suicide

Staff members may experience physical, psychological, and emotional responses that change as time progresses:

‘*every time I shut my eyes all I could see was [the student]*, *so*, *er*, *yeah*, *it did affect us quite badly … the main image is as I’ve pushed the door open that has always stuck with me has that one [describes the physical appearance of the student’s body]*’

Facilities staff.

For some staff, this remained the case even when recalling events during the interview:

***Interviewer***: *Is it ok if I ask you*, *how do you feel about it all when you look back now*?

***Participant***: *[long pause] ‘I just feel like my chest is heavy–‘*

***Interviewer***: *Mmm*

***Participant***: *‘ erm [pause] very sad [pause]’*

***Interviewer***: *So*, *there’s a physical response*?

***Participant***: *Yeah*, *yeah*, *yeah’*

***Interviewer***: - *alongside the emotional one*?

***Participant***: *‘Yes*.*’ [long pause]’*.

Student facing staff.

There was a sense of waiting to feel, putting off emotional engagement with the event as the tasks or their job were still ongoing:

‘*Yeah*, *no*, *I [hesitantly] literally*, *sort of*, *dealt with that and then I came back to do the nightshift the following night*, *it had just*, *I just had to push on and keep myself busy to stop myself thinking about it … obviously being in the same environment it was kind of a bit difficult to forget it at the same time*’

Facilities staff.

For some, this was done through intense efforts of self-control:

‘*while you are doing your job you’re feeling it*, *but you can’t because your responsibility is to the other people*, *you can’t break down*, *it’s incredibly difficult*, *especially if … there’s people crying in front of you*, *it’s incredibly difficult not to break down*, *but you do it*, *you keep it together*’

Student facing staff.

The processes of reflection and developing an understanding of what happened or a rationale for why it might have happened are prominent. This involves much thinking, reasoning, searching, and sense making:

‘*I think it was*, *it was more*, *afterwards*, *when you suddenly realise that’s a–an 18-year-old*, *19-year-old who*, *who was just got to such a state that they can take their own life*’

Facilities staff.

The most frequently expressed regret by staff on behalf of the student was that of ‘waste’:

‘*there was this feeling of waste*, *you know*, *waste of a young life*’

Academic staff.

Staff members notice and highlight in their minds perceived connections with the student–whom they may never have met before–constructing ‘perceptions of closeness’. These may take the form of similarities in age, gender, or circumstance with close family members:

‘*[they’re] an unknown [person] umm [pause] same age as my grandchild*’

Student facing staff.

This process may be triggered by seeing a photo of the student who died, or hearing personal details about the student:

‘*I’ll tell you what affected me most and what is heartbreaking*, *and it probably will make me cry again*, *is just to see pictures of people*, *and*, *and*, *usually if they are abstract students with a name and a course and things*, *and then you see a load of pictures … and that*, *that*, *I found that*, *I actually found that hard*’

Academic staff.

For some, this process started on seeing the student’s body after their death. It seems that as the student is humanised, a process of identification starts, which develops into a need to understand reasons and meaning behind the student’s death. The construction of perceptions of closeness is accompanied closely by an increasing sense of being impacted by the death.

### Needs and fears

Over time, needs and fears come to the fore. Some are connected with job-roles. A process of questioning, fear of having missed something, or not having done more begins:

‘*there is the feeling of guilt*, *obviously*, *because I did invite them … I remember that in that email*, *to all my tutees*, *not just to [the student]*, *all tutees*, *I said*, *please make an appointment*, *I’m looking forward to seeing you … and then you think*, *I wrote this and you know [they] didn’t come*, *and er*, *yeah*, *you feel like could I have said anything*, *could I have made it more urgent*, *to say well*, *you know*, *you need to come or*, *would [they] have shown up anyway [pause] yeah*, *so [big sigh] yeah*, *to be*, *you know*, *as a personal tutor whether there could have been anything else that I could have done differently*’

Academic staff.

Staff also appear mindful of the impact of a suicide death on other students, particularly those close to the student who died:

‘*it’s hard to see*, *how that event has just kind of*, *in many ways ruined [their] university experience umm*, *in an irreparable way really*.’

Student facing staff.

Other needs and fears focus on not being trained to deal with this kind of situation:

‘*it’s always really challenging when students are kind of*, *and obviously they were distraught*, *erm*, *and I think*, *it’s not something I’ve ever had any training in*, *in all the years I’ve been here*’

Facilities staff.

For some staff, an experience of feeling overlooked or forgotten about throughout the event left them feeling in need of recognition or acknowledgement:

‘*it’s like actually when I think back*, *it was*, *my frustration was the fact that people were just kind of almost making that*, *‘well it’ll just happen*, *because they’ll just do whatever needs to be done’*, *you know*, *which we did … but*, *bit of acknowledgement for the team would have been … would have made a big difference*’

Facilities staff.

### Experiences of support

Participants made a range of choices in relation to accessing support:

‘*there obviously wasn’t a great deal of help come from them*, *literally*, *there was an email sent through*, *erm*, *there’s a helpline number if you need it ring it*. *Who are these people*? *You know*, *I’m not gonna randomly talk to somebody over the phone that doesn’t know me or anything like that*’

Facilities staff.

They described different experiences in relation to support accessed or offered:

‘*so*, *I did meet with someone for an hour and a half*, *a couple of hours and just talk through*, *and she was really useful in helping me to reframe why I felt so responsible and actually quite practical about what I could do to make sure that responsibility was shared out*’

Academic staff.

‘*so we were all going to get in a room and talk about [pause] support each other*, *but it didn’t really happen*’

Student facing staff.

Support needs were formed by participants’ individual experiences of the event, their role in responding, and the culture of support within their team and organisation:

‘*I spent a lot of my time checking in that everybody else was ok*’

Facilities staff.

The ways staff processed and understood the event might be moulded by the expectations of their job-role:

‘*but really*, *genuinely because you have to go back to work and into it the next day I think that knowing you’ve got those colleagues around you that you can talk to and you can share and everyone’s kind of going through something similar*, *it’s really important*’

Senior leader.

Cultural and personal factors can shape experiences, perceptions, and potential for a healthy process of reconciliation with the event, and the process of moving forward professionally and personally.

### Personal stories

Experiences of responding and experiencing were influenced or complicated by the personal stories of the staff member. This is about ‘being human’ within the context of the event. These include things in the participant’s personal life and history, including previous experiences with suicide, sudden death, or recent loss of a person close to them:

‘*I’ve got personal history of somebody committing suicide*, *it was somebody I was in a relationship with at the time*, *erm and that kind of*, *that bought stuff back for me on a personal level*’

Facilities staff.

Personal traits informed the felt impact of the event. For instance, some staff take a ‘pro-active’ lead in seeking out support, whereas others wait to be supported:

‘*But it’s important*, *practice what you preach*, *there’s no point saying this is what you should do and then I’m not showing that I can do that*, *er*, *yeah*, *if you don’t look after yourself*, *you won’t be able to look after other people*’

Student facing staff.

One means of self-supporting was by calling on established strategies or personal networks, the things that staff members knew had worked in the past or that did them good:

‘*I’ve got a really good family*, *so I’ve got the support from them as well*’

Facilities staff.

### Cultural stories

Experiences of staff are shaped by the cultural stories within which the event and the response is happening. These cultural stories include structures of response within the HEI; collective attitudes and ideas about student suicide as an event at the particular HEI; cultures of work at the HEI regarding communication, collaboration, and support; individuals’ ideas about suicide, responses, being part of a team, roles and expectations:

‘*and that asking for help is something that we all should do and not feel that we can’t*, *but then I guess part of that is the culture that you create*’

Student facing staff.

‘*it’s not a criticism*, *it’s just how*, *the university’s not set up*, *as you probably know*, *the university’s not set up as a crisis team*, *it’s not set up as a mental health team*, *it’s not set up as a support service really*, *it is set up as an academic institution with student support to help students in their academic life*’

Academic staff.

All of this sits within a wider cultural set of beliefs and ideas about suicide in general, and student suicide in particular–ideas that are influenced by societal behaviours and belief systems; religious beliefs, media messages and responses to suicide and student suicide.

### A grounded theory

Participants evidenced a tension between the categories of ‘Responding to a student suicide’ and ‘Experiencing a student suicide’. There is an entanglement of the ‘professional’ and the ‘human’, between the tasks and the self, between the needs of others and needs of self. Whilst staff acknowledged their need to emotionally process the event, they often did not feel they had time or space to do so. At the core of this tension appears to lie a fierce sense of professionalism within a culture that prioritises the needs of students over the needs of staff. The experiencing of a student suicide for staff members is entangled with undertaking tasks following the suicide; it is not the tasks themselves that cause emotional responses; nor a student death by suicide; rather, it is the entanglement of undertaking tasks within the context of a suicide.

This complexity of ‘Responding’ and ‘Experiencing’ gives rise to ‘Needs and fears’, these frame individual staff members’ responses to the suicide. Fears are often in relation to others, both other students and staff in other job-roles. Additional fears arise from processes of self-scrutiny and checking that nothing gets missed. Needs and fears generate steps toward support and influence staff engagement with, and experiences of, support.

The experiences of responding, experiencing, having needs and fears, and support are further informed by the two remaining categories. ‘Cultural stories’ and ‘Personal stories’ describe the contexts within which staff members are living their experiences. Internal ‘personal’ contexts, including past experiences with suicide and grief, form responses and needs. Staff members ability to be self-supporting and to be able to call on personal strategies and networks forge their capacity for coping and recovery. External ‘cultural’ contexts also mould experiences. These are cultures that exist within the HEI: cultures of caring, community, responsibility, or power. Beyond the HEI at a societal level commonly held beliefs about suicide, or student suicide in particular, further shape staff members understanding of what has happened. Personal stories frame the experiencing of the event and cultural stories mould the context within which that experiencing happens–which, in turn, is itself ‘experienced’.

## Discussion

### Experiencing a student death by suicide

Initial responses to a student suicide included panic, followed by emotional responses including shock, sadness, and bewilderment. Ongoing physiological responses such as sleeplessness and psychological responses such as intrusive imagery associated with the suicide, were experienced by staff members across different job-roles. These adverse experiences are aligned with those reported by other groups of practitioners [14,16] and highlight the need for postvention support for staff within HEIs.

Processes of questioning, reflecting on their own role or responsibilities, and fears of having failed the student, were evident for some participants. This finding aligns with experiences of fear, self-scrutiny, blame, professional doubt, rumination, and fear of legal consequences amongst health practitioners [14,15]. Participants described processes of reflection, which formed part of their search for understanding and meaning around why the student had died. The search for meaning has been evident as part of the grief process for those bereaved by suicide [45,46]. The findings of the current study demonstrate that the search for meaning is evident amongst HEI staff in the wider network of people exposed to and affected by, but not necessarily bereaved by, a suicide death.

The breadth and impact of crisis response tasks undertaken by HEI staff following a student suicide exhibit similarity with those undertaken by first responders. Security staff became involved with tasks that stretched beyond their usual job-role, including performing CPR or managing distressed responses of other students present, such as housemates. Ambulance staff described a similar experience of feeling stretched beyond their usual job-role in having to inform people of the death of a loved one or preserving a potential crime-scene [23]. Knieper [47] affirms that there is a risk of trauma to an individual who witnesses a death or discovers a body following death. The security personnel in this study who were first responders at a student suicide reported experiences of flashbacks and uninvited imagery following the event, together with sleeplessness and anxiety around performing certain work duties such as ‘safe and well’ checks. These findings position the experiences of first responding HEI staff members alongside those of professional first responders, who, despite being specifically trained for and supported in their role, continue to experience struggles when faced with incidences of suicide [23]. Student Minds [48] advocate for attention to be paid to the wellbeing of HEI facilities staff who are often on the front line in noticing, reporting, and responding to student wellbeing crises [48]. The findings of the current study further strengthen calls for skills training and postvention support for this specific and often over-looked group of HEI staff members.

### Personal stories

Participant accounts evidenced diverse support needs. Some seemed well equipped to take steps toward accessing support, utilising existing networks in their personal and professional lives, or by drawing on existing self-care strategies. Others appeared to be waiting for support to be offered, or unable to engage with support available. This diversity of responses may be explained by differences in individual traits, previous life experiences, and existing social supports.

Some participants reported that they chose not to engage with support that was offered, despite feeling unsupported. Similar inconsistencies in accessing support even when it is available have been reported for those who are bereaved following suicide [49,50] and amongst practitioners [7,51]. This may in part be aligned with broader beliefs regarding mental health help-seeking. For instance, attitudes toward mental health specialists are a strong indicator of help-seeking in suicide bereaved adults [52]. Individual differences in terms of proactive traits [53,54] might also offer some explanation for differences in help seeking behaviours. Participants who proactively sought support opportunities were seeking help that would promote a sense of feeling better or facilitate working toward healing from the impact of the suicide. First responders have been found to adopt ‘approach’ or ‘avoidance’ strategies toward help seeking that affect their experiences of coping [55]. Approach strategies require purposeful engagement of effort in understanding and processing experiences, physically and psychologically. Participants who purposefully sought help appeared open to facing and talking explicitly about the discomforting emotional and psychological aspects of the event. Others who reported that they did not seek help, appeared more reluctant in interviews to discuss emotional aspects of the event. It might be that these individuals were using avoidance strategies as a means of evading engagement with threatening feelings or stimuli [55]. Although avoidance strategies may be utilised as a means for self-preservation, this strategy may ultimately result in negative consequences, including increased psychological difficulties and decreased ability to engage with positive experiences [24,55].

Previous experiences of suicide, sudden, or recent death, also affected responses to the student suicide. Participants shared accounts of their previous personal or professional experiences of suicide or a recent bereavement. They articulated connections between past events and the current experience and reported triggering of memories or feelings attached to a previous experience. Cerel et al. [56], stated that individuals may feel affected by a suicide due to previous experiences of suicide; perceiving a cumulative impact. Findings from this study indicate that exposure to a recent bereavement from other causes may also contribute to perceptions of heightened impact. In terms of meeting needs, this suggests that support providers may need to be cautious about making assumptions regarding impact of a student suicide on staff members. Staff members may wish to maintain their privacy around previous experiences of suicide and bereavement, and consequently, reasons underlying their heightened perception of impact may remain unknown to those working alongside or supporting them.

Links to social supports also influence capacity to cope. Informal support utilising pre-existing personal support networks was reported as helpful, and in some cases, served as the main source of support. Increased social support has been shown to lower levels of distress [55] and increase resilience [57]. Personal networks may affect individuals’ abilities to cope and process after the event where those who are unable to draw on social support networks are at greater risk of PTSD following a traumatic event [58].

### Perceptions of closeness and belonging

Throughout participant accounts, their stories spoke of a sense of knowing the student who died and a feeling of closeness to them, irrespective of whether the student was previously known to them. Narratives of safeguarding or protection, or even of feeling in ‘loco parentis’ toward students, evidenced a sense in which the student was felt to matter to participants. In seeking to understand the student’s death, participants engaged in a process of constructing stories and explanations. This took various forms, including relating the student to a family member of a similar age or gender; a family member or close friend who is troubled, distressed, or deceased by suicide; or calling on the experience of being a parent to develop an empathic sense of ‘knowing’ the young person. As such, participants were constructing perceptions of closeness [5] to the student who had died. The concept of ‘perceptions of closeness’ has been previously applied to those who had a pre-existing relationship with the person who died, where a greater perception of closeness is associated with greater perceptions of impact [5]. This study evidences that those who had no prior relationship with the person who died may also perceive closeness. This construction of closeness may, likewise, result in a greater perception of impact. It might be that through this constructed sense of knowing the student, participants created a place and a reason to grieve.

Findings evidence a sense that staff members, across all job-roles, cared about what happened to their students, and felt a sense of responsibility for students’ wellbeing. The teacher-student relationship in HEIs has been described as multi-dimensional, consisting of ‘closeness, care, connection, safety, trust, honesty, fairness, respect, openness, support, encouragement, availability and approachability’ [59] (p. 378). The idea of ‘caring’ for students is regarded as a humanistic value and a moral responsibility, although the concept has received little attention in the literature [59].

Belongingness describes the need to belong as being a fundamental human motivation [60]. To experience belongingness, people need frequent personal contact with others within the context of a bond or relationship marked by stability, concern, and continuation into the foreseeable future. In a study of nursing students, it was found that their experiences of the staff-student relationship were key to the students’ sense of belongingness [61]. For participants in this study, the question is raised as to whether perceptions of caring, safeguarding, and responsibility; and the process of constructing perceptions of closeness arise from underlying, shared narratives, of belonging to the university.

### Cultural stories

The experiences of those in health, social care, and education job-roles following a death by suicide have been shown to be socially and culturally situated [13]. Throughout these participant accounts there was a palpable sense of shared community as being the context within which experiences, needs, and perceptions were situated. Mcmillan and Chavis [62] defined and theorised the concept of sense of community which may be applicable to a HEI setting. They identified four elements (membership; influence; integration and fulfilment of needs; and shared emotional connection) that, when experienced together, nurture a sense of community within or across a group of individuals who may be linked geographically or relationally.

Within this study members of staff who are usually responsible for security, student wellbeing, senior management tasks, and chaplaincy, all became first responders in a moment of crisis. They were responding on behalf of a student who was a part of their HEI community. This factor differentiates their experiences from those of community-based first responders, such as paramedics and police officers. For HEI staff, there is the complexity of being the responder, whilst sharing the commonality of community with the deceased. This situation is not evident in the literature for any other first responder or practitioner and appears unique to a HEI setting. It is plausible that narratives of community and culture present in participant accounts are evidence that a strong sense of community exists within which the sense of belongingness and perceptions of closeness are nurtured and strengthened. It is indeed the nature of the HEI as an organisation within which community and belongingness are integral, that may further explain the heightened perceptions of impact following a student suicide.

Wider cultural ideas of suicide may not have been explicitly stated in participants’ accounts, but implicit in their words was the idea that a death by suicide was felt to be somehow worse than a student death by other means. This implicit message was present in the sense of bewilderment and horror expressed that a person so young would choose to end their own life. It was also present in the fears shared about the potential impact on institutional reputation, and perceived fears about suicide clusters and other student’s wellbeing and safety. These fears differentiate HEI staff experiences from other groups of workers. They may be valid fears given there is known reporting bias around the topic of suicide, where some suicide deaths are more frequently and widely reported than others [63]. Female suicide, particularly young females, and suicides of students are disproportionately reported in print and online news reports in the UK and Republic of Ireland [63].

A suicide cluster occurs when there are more suicide deaths than expected in time or place, or both [64]. Clustering of suicidal behaviour is more frequent in young people and is likely to occur in an institution such as a university [64]. There have been recent suicide clusters in several UK HEIs. Participants in the current study held the perception that media reporting of student suicide was a causal factor contributing to HEI reputational damage. However, the relationship between media reporting and potential reputational damage of HEIs remains unexplored. Evidence from these participant accounts suggest that they were concerned about misperceptions amongst members of the public regarding rates of student suicide being higher than those of their age-matched peers in the general non-student population. This misperception erroneously identifies HEIs as unsafe environments for young people. Recent research suggests the incidence of student suicide per 100,000 is less than half of those of age matched non-students in the general population [65].

## Strengths and limitations

This is the first study to explore the experiences of staff in UK HEIs following a student death by suicide. This exploration has generated novel findings that inform current knowledge regarding the impact of a student suicide within UK HEIs and, more broadly, the impact of suicide on wider networks around a person who dies by suicide. The findings inform future development of postvention support for UK HEI staff following a student death by suicide and postvention needs and provision amongst wider populations, including staff working in secondary and further education (FE), other residential communities, or vocational training settings. Whilst further research is needed to understand causation, relationships, and longitudinal outcomes, these findings can inform subsequent research that seeks to further explore the impact of student death by suicide in UK HEIs. This study specifically focused on HEIs in the UK and findings may not be completely transferable internationally due to differences in culture and processes.

Grounded Theory was particularly suited to this un-researched topic and population [41]. Theory was developed inductively from the experiences of participants. In combining survey and interview data for the grounded theory analysis, a robust theoretical construction was developed. Two factors impinged on the processes of data collection and analysis applied. First, the small sample size. Often a process of theoretical sampling is employed in grounded theory, whereby, participants are selected to answer questions arising through the analytic process [41]. The finite sample prevented this process; however, the qualitative survey data enabled the processes of checking and comparison within the construction of categories and theory. Second, interviews were undertaken in scheduled blocks. Data collection and analysis in grounded theory would usually take the form of interview–transcribe–code–interview–transcribe–code and so on [41] to allow opportunity for subsequent interviews to either check early findings, or to fill apparent gaps. To overcome this challenge, extensive note making after each interview captured initial reflections and impressions and informed the development of the interview topic guide. Interviews were transcribed and coded at the earliest opportunity following each block of interviews. So, for this study, the process took the following pattern; interview–notes–interview–notes–transcribe–code–transcribe–code–interview–and so on. This pattern ensured that as data were coded, subsequent interviews were informed by knowledge gained from the coding process. It is also worth noting that two participants were interviewed by telephone and eight in face-to-face interviews. The same topic guide was utilised, and the data gathered from participants by telephone shared similar depth and breadth of detail as that gathered from face-to-face interviews.

## Recommendations for research and practice

The findings of this study inform the future provision of postvention support to staff in UK HEIs after a student death by suicide. Further research exploring the experiences of other affected groups including close friends, student peers, and family members would provide evidence to inform the development of holistic postvention guidance for the UK HEI and FE sectors.

Further research might ask how can we best support staff who, due to their roles, are focussed on supporting others before themselves; and how can we encourage staff to engage with support and activities that might meet their own needs? Research is needed to establish whether, in constructing a perception of closeness, the individual does experience a greater perception of impact, as has been found for those with a pre-existing relationship with the deceased [66]. Current research in the area of postvention pays attention to processes of grief and loss; future research might explore experiences of trauma amongst those in wider networks who take on specific tasks following a death by suicide..

Current postvention provision with UK HEIs has been designed and is being delivered without a context-specific evidence base. This study provides evidence that underpins the following recommendations for postvention support for staff members:

- Responsibility for sharing the news of a student suicide to wider groups of student peers should be undertaken by a trained member of staff who understands the potential risks involved when talking about or sharing information about a death by suicide;
- It is imperative for the safety and wellbeing of other students that language and messages around suicide are shared appropriately and safely;
- Postvention support policies within HEIs should include acknowledgement that staff members may need support whether or not they knew the student who died, and should include specific trauma-focused support to staff members who are involved in crisis response tasks;
- HEI provision should align support offered with that being sought elsewhere by the individual; and combine formal and informal support offers to meet individual needs;
- Support should be offered pro-actively and repeatedly over the subsequent days, weeks, and months.

## Conclusion

This study describes previously unexplored experiences of HEI staff members following a student death by suicide. Findings evidence adverse emotional responses and trauma symptoms, together with ongoing impact on work practice. Experiences are informed by staff members sense of connection with and ‘knowing’ the student who died. Specific, unique aspects of the sense of community within HEI setting may shape the experiences and impact for staff members. This study provides strong evidence of the complexity that tasks and additional roles or responsibilities introduce for staff, affecting their emotional processing following a suicide in the workplace. Other complicating factors include previous experiences of suicide or bereavement, and availability of social support networks. Experiences reported by staff members incorporate aspects previously reported by other groups of professional workers, including first responders, and those bereaved by suicide. It is evident for this HEI staff population, those who perceive themselves to be exposed or affected by suicide may share post-event experiences similar to those who are bereaved. These findings inform the conclusion that HEI staff members require bespoke postvention support specifically tailored to respond to the range and complexity of HEI staff needs and informed by knowledge of the HEI social and cultural context.

## Supporting information

S1 Table Interview topic guide. (DOCX)

S2 Table Summary of focused codes, sub-categories, categories and core category. (DOCX)
