# Supplementary material for: Bearing witness: A grounded theory of the experiences of staff at two United Kingdom Higher Education Institutions following a student death by suicide
Source: PLoS One. 2021 May 12;16(5):e0251369. doi: 10.1371/journal.pone.0251369 (PMC8115842; doi:10.1371/journal.pone.0251369)
Supplement: S1 Table — (DOCX) [file pone.0251369.s001.docx]

**S1 Appendix: Interview Topic Guide**

It is usual in grounded theory interviewing that the topic guide is an open and developing tool. As knowledge is gained from participant data so amendments or additions are made to the topic guide such that a process of checking and testing findings can be incorporated into the ongoing data collection process. In this study, the topic guide was updated on two occasions, as detailed below.

**Topic Guide – Version 1 – Used for Interviews 1 & 2.**

| **Topic** | **Potential Question** |
| --- | --- |
| Participant’s experiences and perceptions of events following the student death by suicide | I would like to start our interview by asking you if you can tell me about the events that followed the student’s death by suicide that happened here in [month and year]?  Could you tell me about your thoughts and feelings following the suicide?  Tell me about any ways in which it affected you?  What kinds of things did you need to help you at that time?  What kinds of support were you offered?  Can you tell me about something that was particularly helpful at that time?  What other things might have been helpful to you? |
| Participant’s experience and perception of taking on any role/s following the student death by suicide | Tell me about any particular tasks or jobs that you took on following the suicide?  What was it like for you to undertake that role/task/job?  What helped you to manage that role/task/job?  What were the difficulties that you encountered in doing that role/task/job? |
| Participant’s experience of helpfulness following the student death by suicide  Additional experiences and perceptions not covered within the interview so far | Is there any person or organisation that has been particularly helpful or supportive of you since the student’s death? What kind of things have they done?  Is there something that you might not have thought about before that has occurred to you during this interview?  Is there something else that you think I should know about to understand your experiences better? |

**Topic Guide – Version 2 – Used for Interviews 3 – 6**

**Additions made to version 1**

Opening Questions

These were added to the topic guide as I found myself asking them from the first interview as a way to settle the participant and help them to feel comfortable in talking with me.

So we are here to talk about your experiences following a student death by suicide here at the University, is it ok if I ask you some questions about that now?

This question was added as I found myself using a similarly phrased opening to check that the participant was ready to move onto talking about the main topic

How did you hear about the student’s death?

This question was added in response to my experience of hearing the first two participants tell me their ‘stories’. In both instance this was their starting point.

How did you know what to do?

This question was added in response to hearing participants’ accounts of feeling unsure about what they should do.

| **Topic** | **Potential Question** |
| --- | --- |
| Opening questions | I would like to start our interview by asking you if you can tell me what your job is and what kinds of things you do  How long have you worked here? Have you always done the same job here?  What would a typical day look like? |
| Participant’s experiences and perceptions of events following the student death by suicide | So we are here to talk about your experiences following a student death by suicide here at the University, is it ok if I ask you some questions about that now?  How did you hear about the student’s death?  What were the events that followed the student’s death by suicide?  Could you tell me about your thoughts and feelings following the suicide?  Tell me about any ways in which it affected you?  What kinds of things did you need to help you at that time?  What kinds of support were you offered?  Can you tell me about something that was particularly helpful at that time?  What other things might have been helpful to you? |
| Participant’s experience and perception of taking on any role/s following the student death by suicide | Tell me about any particular tasks or jobs that you took on following the suicide?  How did you know what to do?  What was it like for you to undertake that role/task/job?  What helped you to manage that role/task/job?  What were the difficulties that you encountered in doing that role/task/job? |
| Participant’s experience of helpfulness following the student death by suicide  Additional experiences and perceptions not covered within the interview so far | Is there any person or organisation that has been particularly helpful or supportive of you since the student’s death? What kind of things have they done?  Is there something that you might not have thought about before that has occurred to you during this interview?  Is there something else that you think I should know about to understand your experiences better? |

**Topic Guide – Version 3 – Used for Interviews 7 - 10**

**Additions made to version 2**

Tell me about any ways in which it continues to affect you.

This question was added in response to hearing participants accounts of ongoing impact

Can you share with me any particular moment or image that has stayed with you?

This question was added after one participant expressed surprise that I hadn’t asked about trauma or used the word trauma – she talked vividly of imagery and the impact it had on her. On checking the transcripts of other participants I found that accounts of imagery and ‘flashbacks’ were present in most transcripts.

Tell me about any ways in which the experience has changed the way you work now.

This question was added after a participant talked repeatedly about how they had changed their work practice. On checking previous transcripts, I found other participants had spoken about doing things differently now.

How do you feel about it all, when you look back now?

This question was added in response to seeing the ‘in the moment’ emotional responses that participants were displaying during the interviews. It was clear that this event continued to carry emotional resonance. The question provided a way of acknowledging and validating their ‘in the moment’ responses, whilst opening an opportunity for clearer articulation of what they were feeling in the moment.

| **Topic** | **Potential Question** |
| --- | --- |
| Opening questions | I would like to start our interview by asking you if you can tell me what your job is and what kinds of things you do  How long have you worked here? Have you always done the same job here?  What would a typical day look like? |
| Participant’s experiences and perceptions of events following the student death by suicide | So we are here to talk about your experiences following a student death by suicide here at the University, is it ok if I ask you some questions about that now?  How did you hear about the student’s death?  What were the events that followed the student’s death by suicide?  Could you tell me about your thoughts and feelings following the suicide?  Tell me about any ways in which it affected you?  What kinds of things did you need to help you at that time?  What kinds of support were you offered?  Can you tell me about something that was particularly helpful at that time?  What other things might have been helpful to you?  Tell me about any ways in which it continues to affect you.  Can you share with me any particular moment or image that has stayed with you? |
| Participant’s experience and perception of taking on any role/s following the student death by suicide | Tell me about any particular tasks or jobs that you took on following the suicide?  How did you know what to do?  What was it like for you to undertake that role/task/job?  What helped you to manage that role/task/job?  What were the difficulties that you encountered in doing that role/task/job?  Tell me about any ways in which the experience has changed the way you work now. |
| Participant’s experience of helpfulness following the student death by suicide  Additional experiences and perceptions not covered within the interview so far | Is there any person or organisation that has been particularly helpful or supportive of you since the student’s death? What kind of things have they done?  How do you feel about it all, when you look back now?  Is there something that you might not have thought about before that has occurred to you during this interview?  Is there something else that you think I should know about to understand your experiences better? |
