# Supplementary material for: Bearing witness: A grounded theory of the experiences of staff at two United Kingdom Higher Education Institutions following a student death by suicide
Source: PLoS One. 2021 May 12;16(5):e0251369. doi: 10.1371/journal.pone.0251369 (PMC8115842; doi:10.1371/journal.pone.0251369)
Supplement: S2 Table — (DOCX) [file pone.0251369.s002.docx]

**S2 Appendix: Summary of focused codes, sub-categories, categories and core category**

| **Core Category** | **Category** | **Sub-Category** | **Focused Codes** |
| --- | --- | --- | --- |
| Bearing witness  Bearing Witness | Responding to a student death by suicide | Being the responder | Finding the body |
|  |  |  | Seeing the body |
|  |  |  | Hearing the news |
|  |  |  | Crisis response tasks |
|  |  |  | Strategy, Comms & Management tasks |
|  |  |  | Support tasks |
|  |  |  | Practical & Administrative tasks |
|  |  |  | Managing student needs |
|  |  |  | Supporting other students |
|  |  |  | Containing the narrative |
|  |  |  | Offering support to staff |
|  |  |  | Strategies for support |
|  |  | Ways of responding | Doing the job |
|  |  |  | Challenges of responding |
|  |  |  | Being directed |
|  |  |  | Being a leader |
|  |  |  | Process & Procedure |
|  |  |  | Working with other agencies |
|  |  |  | Information sharing |
|  |  |  | Roles & Expectations |
|  |  |  | Team working |
|  |  |  | Working together |
|  | Experiencing a student death by suicide | Body & Mind | Physical responses |
|  |  |  | Initial responses |
|  |  |  | Emotions on hold |
|  |  |  | Emotional responses |
|  |  |  | Visual echoes |
|  |  |  | Managing the emotions |
|  |  | Reflections and Perceptions | After the event |
|  |  |  | ‘an impactful event’ |
|  |  |  | Recalling |
|  |  |  | Wondering why |
|  |  |  | Searching for meaning |
|  |  |  | Ongoing thoughts |
|  |  |  | Finding personal connection |
|  |  |  | Leaving a note |
|  |  |  | The unknown student |
|  |  |  | Knowing the student |
|  | Needs & Fears | | Impacting on other students |
|  |  |  | Reputation & Blame |
|  |  |  | Unmet needs |
|  |  |  | Dealing with needs |
|  |  |  | Training needs |
|  |  |  | Needing acknowledgment & recognition |
|  | Experiences of Support | | Accessing support |
|  |  |  | Engaging with support |
|  |  |  | Experiencing support |
|  |  |  | Not being supported |
|  |  |  | Building relationships with students |
|  |  |  | Experiences of supporting |
|  |  |  | Supporting each other |
|  | Personal stories | | Calling on experience |
|  |  |  | Supporting self |
|  |  |  | Using strategies |
|  |  |  | Using networks |
|  |  |  | Human connections |
|  |  |  | Not/the first time |
|  |  |  | Reflecting on the experience |
|  |  |  | Emotional responses & reflections during interview |
|  | Cultural Stories | | Organisational provision |
|  |  |  | Cultures of support |
|  |  |  | Creating culture |
